# Paradoxical Worsening of Cerebral Venous Sinus Thrombosis Due to Heparin-Induced Thrombocytopenia: A Case Report and Literature Review

**DOI:** 10.7759/cureus.58124

**Published:** 2024-04-12

**Authors:** Gento Moriguchi, Toru Umehara, Yoshihiro Yano, Toshiaki Fujita, Haruhiko Kishima

**Affiliations:** 1 Department of Neurosurgery, Hanwa Memorial Hospital, Osaka, JPN; 2 Department of Neurosurgery, Osaka University Graduate School of Medicine, Suita, JPN

**Keywords:** cerebral venous infarction, tonic convulsive seizures, acute exacerbation, heparin induced thrombocytopenia (hit), cerebral venous sinus thrombosis (cvst)

## Abstract

Development of cerebral venous sinus thrombosis (CVST) is a rare manifestation of heparin-induced thrombocytopenia (HIT). Herein, we present a case in which heparin administration for primary CVST caused paradoxical worsening of CVST secondary to HIT. A 53-year-old woman diagnosed with CVST was provided with intravenous unfractionated heparin therapy. After 12 days, the patient presented tonic convulsive seizures (TCS). Subsequent magnetic resonance image (MRI) scans revealed an exacerbation of cerebral edema with a subcortical hemorrhage on the left parietal lobe. Laboratory test results revealed a significant decline in platelet count. Heparin was immediately discontinued and replaced with argatroban. The definitive diagnosis of HIT was made through the presence of HIT antibodies. The present case, in which HIT caused the secondary CVST exacerbation, is distinctly rare. Our case provides an instructive example by highlighting the potential of TCS as the first sign of HIT development during CVST treatment.

## Introduction

Heparin-induced thrombocytopenia (HIT) is categorized as type 1 or type II. Type I HIT is a mild transient nonimmune disorder resulting from the direct effect of heparin on platelet activation. Type II HIT is a clinically significant prothrombotic disorder caused by immunoglobulin (Ig)G-specific antibodies directed toward complexes consisting of platelet factor 4 (PF4) and heparin. For simplicity, the term HIT refers to type 2 HIT. Thus, antibody-mediated platelet activation and consequent thrombin generation lead to an increased risk of thrombosis in patients with HIT [1]. HIT has been estimated to occur in 0.1%-7% of patients treated with heparin and the case fatality rate averaged 5%-10% without the correct treatment [2,3]. Especially in patients who have already received heparin administration for primary thrombotic disorders and hypercoagulable states, the development of HIT can lead to paradoxical acute exacerbation of the underlying thrombosis.

Thrombosis occurs in approximately 50% of patients with HIT who are not treated with an alternative non-heparin anticoagulant, with venous thrombi being more common than arterial thrombi [4,5]. Although venous thromboembolism (VTE), including pulmonary embolism and deep vein thrombosis, are the most common complications [6], development of cerebral venous sinus thrombosis (CVST) is a rare occurrence in patients with HIT, with relatively few reports currently available in the literature [7,8].

Here, we presented a rare and instructive case in which HIT caused paradoxical worsening of CVST after heparin administration for primary CVST. Since this clinical sequelae, HIT-associated CVST exacerbation (HACE), is currently not well recognized owing to its rarity, we explored the clinical features and management of the HACE with a review of previous reports.

## Case presentation

**Figure 1:**
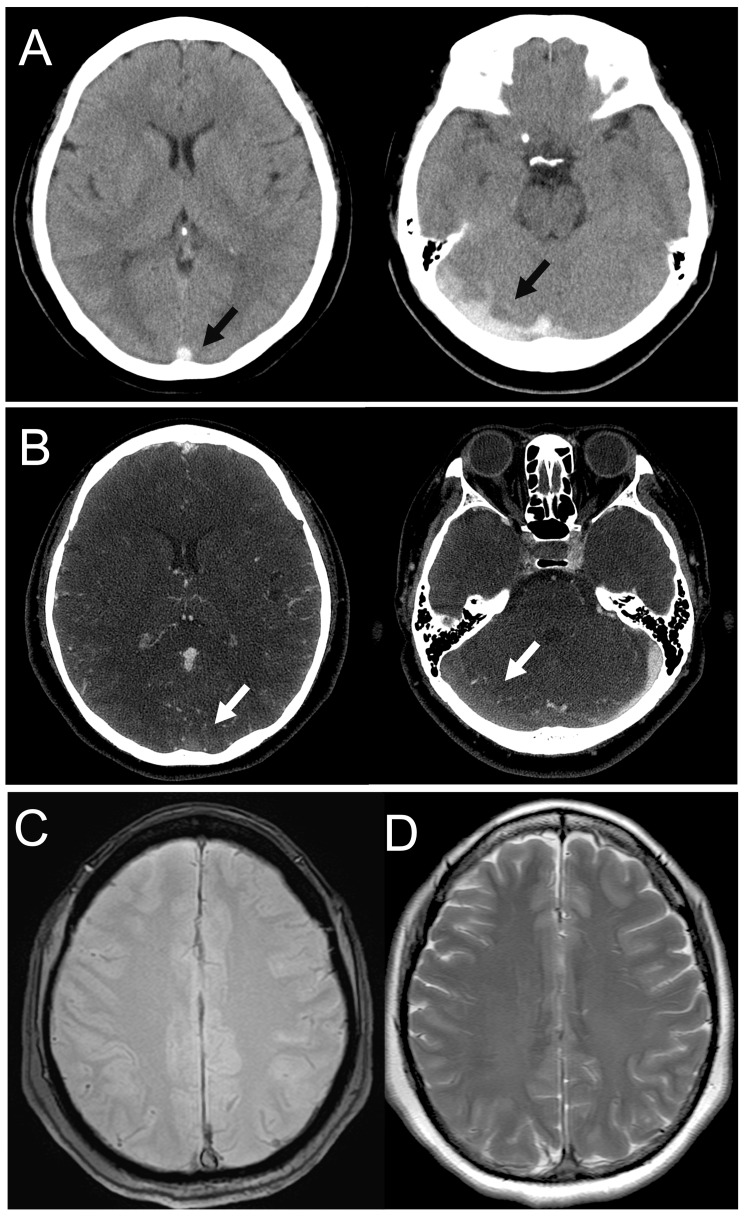
Imaging of CVST on admission Axial CT scans (upper row), CT venography (middle row), and MRI images (bottom row) obtained upon admission are shown. A dense clot sign (black arrows) and a filling defect (white arrows) in the superior sagittal and right transverse sinuses were suggestive of CVST (A, B). MR imaging at the supratentorial level shows no cerebral edema and intracranial hemorrhage (C: T2*, D: T2-weighted image). CVST - cerebral venous sinus thrombosis

A 53-year-old woman with no significant medical history (height, 157 cm; body weight, 62 kg; body mass index, 25.2 kg/m^2^) was brought to our hospital with a two-day history of a throbbing headache and intermittent vomiting; no focal neurological deficits were noted. She had a temperature of 36.3 °C, blood pressure of 189/109 mm Hg, pulse rate of 98 beats/min, and Glasgow Coma Scale score of 15 (E4, V5, and M6). A computed tomography (CT) scan of the head revealed a dense clot sign with a high-density area in the superior sagittal and right transverse sinuses, which is suggestive of thrombosis. Subsequent CT venography identified a filling defect from the superior sagittal sinus to the right sigmoid sinus (Figures 1A-1D). No sign of hemorrhagic venous infarction including edema was detected at that point, whereas laboratory test results revealed an elevated D-dimer level (15.0 µg/mL). As the concluding diagnosis was CVST, the patient was hospitalized for anticoagulant therapy with intravenous unfractionated heparin (UFH) therapy.

The routine screening for antiphospholipid antibody syndrome using anti-cardiolipin β2-glycoprotein I complex antibody and lupus anticoagulant were negative. Levels of coagulation inhibitors, such as antithrombin III, protein C, and protein S, were also within the normal range. The patient had no underlying medical conditions or history of oral contraceptive use. The patient also tested negative for sudden acute respiratory syndrome coronavirus 2 (SARS-CoV-2) using polymerase chain reaction screening on admission; ≥3 months have passed after the final dose of SARS-CoV-2 mRNA-based vaccine, making it less likely for this case to be SARS-CoV-2-related thrombosis.

**Figure 2:**
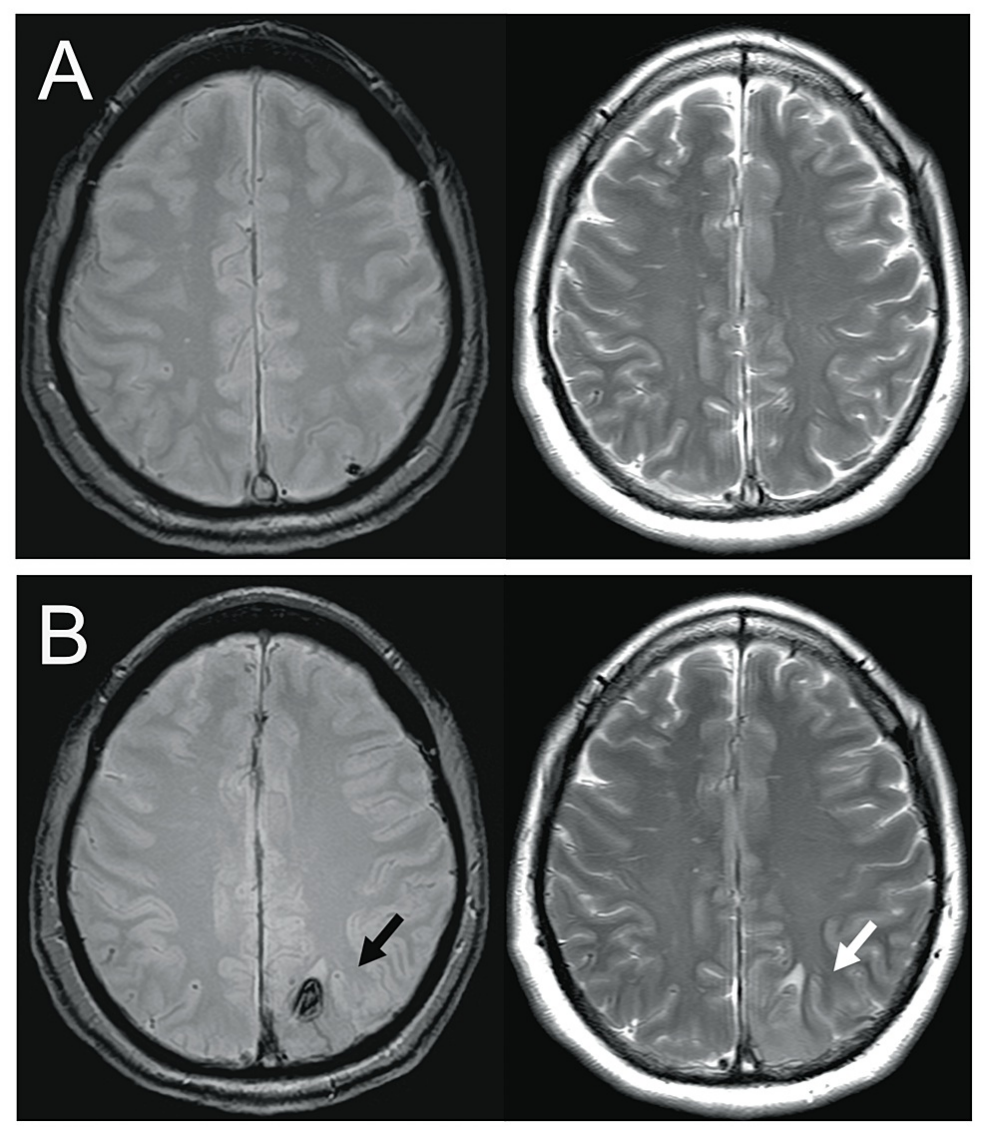
Time series analysis of exacerbation of CVST after heparinization Axial T2* (left) and T2 (right) weighted images on the seventh (A) and 12th day (B) after heparinization for CVST are shown. MRI remained unchanged from the admission assessment without clear evidence of venous infarction (A). However, on the 12th day when HIT developed, MRI revealed subcortical hemorrhage (black arrow) and cerebral edema (white arrow) in the left parietal lobe, suggesting acute exacerbation of CVST (B). CVST - cerebral venous sinus thrombosis, HIT - heparin-induced thrombocytopenia

**Figure 3:**
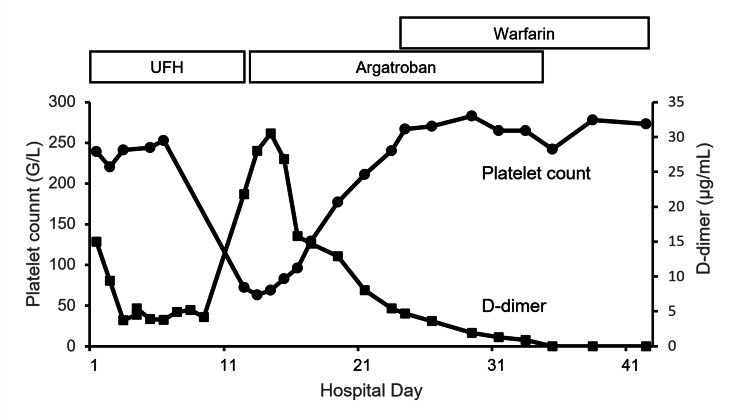
Longitudinal changes of D-dimer levels and platelet counts The graph shows platelet counts (black circles) and D-dimer levels (white squares) during hospitalization. The patient was treated with intravenous UFH from admission to day 12, resulting in a significant decline in platelet count and D-dimer elevation. With clinical suspicion of HIT, UFH was promptly replaced with argatroban. The platelet count nadir was 63 × 10^9^/L on the 13th day, and the peak D-dimer was 30.5 µg/mL on the 14th day. After confirmation that the platelet counts recovered to the baseline thereafter and the D-dimer levels passed a peak, transitioning from argatroban to warfarin therapy was performed. HIT - heparin-induced thrombocytopenia, UFH - unfractionated heparin

**Figure 4:**
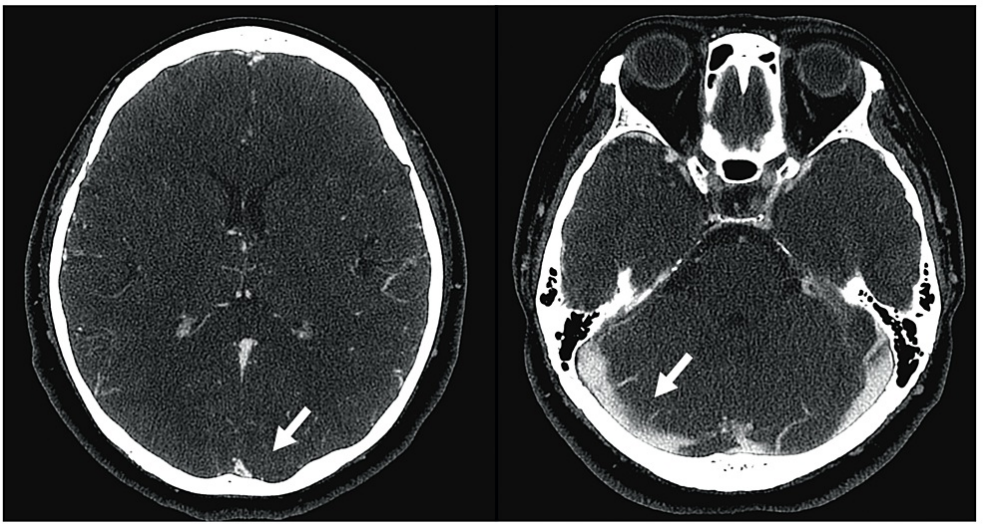
Images after healing of CVST A follow-up CT venography conducted on the 79th day revealed the recanalization of the superior sagittal sinus to the transverse sinus (white arrows). CVST - cerebral venous sinus thrombosis

The patient received the UFH therapy after admission with close monitoring to maintain prolongation of the activated partial thromboplastin time to two to 2.5-fold that of the initial value (24.5 s). Up to the 11th day after hospitalization, the D-dimer had favorably decreased and stabilized at the low level, and the symptoms of headache and nausea gradually improved without neurologic and radiographical worsening. However, on hospitalization day 12, the patient presented with vomiting and tonic convulsive seizures (TCS) that lasted for >5 min until intravenous infusions of diazepam (5 mg) and levetiracetam (500 mg). Subsequent magnetic resonance image (MRI) scans revealed an acute exacerbation of cerebral edema with a subcortical hemorrhage on the left parietal lobe (Figures 2A, 2B). Laboratory test results indicated that the platelet count had significantly dropped to 72 × 10^9^/L, in comparison with 239 × 10^9^/L, which was initial measured upon admission. Additionally, the D-dimer level was significantly reelevated (21.8 µg/mL), although venous leg ultrasound results were negative for deep venous thrombosis. The 4T score reached 7, leading to a suspicion of HIT. Heparin administration was discontinued immediately and replaced with argatroban at a rate of 0.7 µg/kg/min. The latex agglutination assay demonstrated the presence of HIT antibodies from a blood specimen collected on the 14th day (optical density 5.7 U/mL, normal <1 U/mL). Within a few days after the administration of argatroban, the platelet counts recovered to almost the baseline at >200 × 10^9^/L and the D-dimer levels passed a peak. Longitudinal changes in the D-dimer levels and platelet counts during hospitalization are illustrated in Figure 3. As adequate platelet levels had been maintained, transitioning from argatroban to warfarin therapy was performed. On the 34th day, administration of argatroban was discontinued after the prothrombin time-international normalized ratio (PT-INR) reached the therapeutic range. No further seizures occurred after the initial attack, and daily oral administration of levetiracetam 1,000 mg was discontinued a few weeks after the initial seizure. The patient was discharged on the 45th day without any sequelae. The recanalization of the superior sagittal sinus to the transverse sinus was confirmed through follow-up CT venography on the 79th day (Figure 4). The patient has had an uneventful course after hospital discharge without recurrent venous thrombosis for more than six months, and warfarin use was discontinued thereafter.

## Discussion

Complex conditions such as the present case, in which HIT caused the acute exacerbation of CVST after heparinization for primary CVST, are rare. To the best of our knowledge, only five cases of HACE have been previously reported (Table 1) [9-13]. Here, we reported the first HACE case presenting with an epileptic seizure at onset. Although seizures represent a common symptom in patients with CVST, our case provided an instructive example by highlighting the potential of seizures as the first sign of HIT development during the treatment of CVST.

**Table 1:** Summary of HIT-associated CVST exacerbation DC, decompressive craniectomy; EVT, endovascular treatment; F, female; HACE, heparin-induced thrombocytopenia-asscociated cerebral venous sinus thrombosis exacerbation; ICH, intracranial hemorrhage; L., left; LOC, level of consciousness; R., right

Authors & year	Age (years) /sex	Symptoms at HACE	ICH following HACE	Duration of heparin exposure (days)	Platelets nadir (x10^9^/L)	Treatment for HACE	Outcome
Ichihara-Kawase et al., 2010 [7]	36/F	Dysphasia; R.hemiparesis	No	13	60	DC	Dead
Richard et al., 2010 [8]	61/F	Headache; Vomiting	Yes	11	42	Danaparoid; warfarin; DC	R.hemiparesis; aphasia
Hsieh et al., 2013 [9]	67/F	Vertigo; Nausea	Yes	11	65	Fondaparinux	Full recovery
Fukushima et al., 2019 [10]	47/F	R.hemiplegia; Reduced LOC	No	14	108	Argatroban; warfarin; EVT	Full recovery
Khan et al., 2022 [11]	22/F	None	Yes	6	117	Argatroban; apixiban	Full recovery
The present case	53/F	Seizure; Vomiting	Yes	12	72	Argatroban; warfarin	Full recovery

In a review of all reported cases with the HACE, patients were exclusively female, likely because CVST and HIT are more common in women with a ratio of approximately 3:1 and 2:1, respectively [14,15]. Typical-onset HIT commonly manifests five to 14 days after heparin administration. The HACE occurred within this typical onset period in any of these cases. Among six cases of the HACE, four patients manifested intracranial hemorrhage after the onset of HIT, and two required decompression craniectomy (DC) against hemorrhagic venous infarction. Based on systematic reviews regarding HIT-related CVST, it has higher rates of intracerebral hemorrhage and a higher mortality risk than CVST alone, and the most common cause of death was hemorrhagic venous infarction, with a mortality rate of 28.6% during hospitalization [7,8].

Frequent neurological assessments and platelet count monitoring are essential for the early detection of worsening CVST secondary to HIT. However, laboratory tests may not be significant for thrombocytopenia at the onset of the HACE, as reported by Richard et al. [10]. In approximately 25% of patients with HIT, progressive or recurrent thrombosis has been reported to virtually develop prior to a significant decline in platelet count [4]. In essence, physicians should not characterize clinical deterioration of CVST easily as heparin resistance, but instead consider the possibility of HIT, even if the decline in platelet count is slight or undetectable at that point. HIT antibody measurements should help rule out HACE, especially between days 5 and 14 after the initiation of heparin, when HIT typically occurs. No evidence has yet shown whether primary CVST itself is a risk factor for the development of HIT [15]. Alternatively, Hsieh et al. suggested that HIT developed during the treatment of CVST may complicate the thrombus present in the cerebral venous sinus, instead of initiating a new thrombus elsewhere. No findings are suggestive of VTE were observed in the present case, further supporting the findings of Hsieh et al. [11].

Anticoagulant treatment with heparin is still the first choice for patients with primary CVST, even for those accompanied by hemorrhagic stroke; however, frequent neurological assessments and monitoring of platelet counts are crucial as a precaution for paradoxical CVST exacerbation because of HIT. With clinical suspicion of HIT during heparinization for the CVST, patients should be promptly treated with non-heparin anticoagulants after the discontinuation of heparin, even if in the absence of thrombosis progression [16]. For several of these cases, the first line of treatment is the use of argatroban, although there was no significant difference among alternative non-heparin anticoagulants in the incidence of hemorrhagic or thromboembolic events [7,17]. Along with alternative anticoagulants, surgical treatment with DC, or neuroendovascular therapy such as aspiration, balloon sinuplasty, and local thrombolysis were added, as appropriate, for some cases presenting with CVST accompanied by HIT [9,10,12]. Collectively, rapid identification and treatment of HIT-induced CVST is imperative to prevent morbidity and mortality.

## Conclusions

We report a rare case of HACE following heparin therapy. In cases of clinical deterioration such as seizures during CVST treatment with heparin, physicians should consider the possibility of the development of HIT. While heparin remains the first choice for patients with CVST, frequent neurological assessments and monitoring of platelet counts are essential as a precaution for the potential of paradoxical CVST occurring exacerbation secondary to HIT.
